# A comparison of survey method efficiencies for estimating densities of zebra mussels (*Dreissena polymorpha*)

**DOI:** 10.7717/peerj.15528

**Published:** 2023-07-10

**Authors:** Jake M. Ferguson, Laura Jiménez, Aislyn A. Keyes, Austen Hilding, Michael A. McCartney, Katherine St. Clair, Douglas H. Johnson, John R. Fieberg

**Affiliations:** 1 School of Life Sciences, University of Hawaii at Manoa, Honolulu, Hawaii, United States; 2 Department of Fisheries, Wildlife, and Conservation Biology, University of Minnesota—Twin Cities Campus, St Paul, Minnesota, United States; 3 Minnesota Aquatic Invasive Species Research Center, University of Minnesota—Twin Cities Campus, St Paul, Minnesota, United States; 4 Department of Ecology and Evoluationary Biology, University of Colorado at Boulder, Boulder, Colorado, United States; 5 Department of Mathematics, Carleton College, Northfield, Minnesota, United States

**Keywords:** Abundance estimation, Aquatic invasive species, Detection probability, Distance-removal survey, Quadrat survey, Removal survey, Underwater visual survey

## Abstract

Abundance surveys are commonly used to estimate plant or animal densities and frequently require estimating detection probabilities to account for imperfect detection. The estimation of detection probabilities requires additional measurements that take time, potentially reducing the efficiency of the survey when applied to high-density populations. We conducted quadrat, removal, and distance surveys of zebra mussels (*Dreissena polymorpha*) in three central Minnesota lakes and determined how much survey effort would be required to achieve a pre-specified level of precision for each abundance estimator, allowing us to directly compare survey design efficiencies across a range of conditions. We found that the required sampling effort needed to achieve our precision goal depended on both the survey design and population density. At low densities, survey designs that could cover large areas but with lower detection probabilities, such as distance surveys, were more efficient (*i.e*., required less sampling effort to achieve the same level of precision). However, at high densities, quadrat surveys, which tend to cover less area but with high detection rates, were more efficient. These results demonstrate that the best survey design is likely to be context-specific, requiring some prior knowledge of the underlying population density and the cost/time needed to collect additional information for estimating detection probabilities.

## Introduction

In Ferguson et al. (2019), we explored the application of distance sampling for estimating the abundance of zebra mussels (*Dreissena polymorpha*) in newly infested lakes. In contrast with conventional distance sampling (Buckland et al., 2001), we found that multiple observers were needed to account for imperfect detection on the transect line. A secondary result was that our observers had significantly different detection probabilities. When discussing these results, one observer reported being more focused on surveying quickly and covering more area while the other observer reported going more slowly to ensure high detection probabilities. This led us to wonder about a potential tradeoff between surveyor speed and detection probability. Specifically, when is it better to survey slowly and deliberately, resulting in high detection rates but at the cost of covering less area, or to survey more quickly to cover more area but at the cost of lower detection rates?

Much work has gone into comparing alternative survey designs in specific systems (*e.g*., Samoilys & Carlos, 2000; Norvell, Howe & Parrish, 2003; Pooler & Smith, 2005). Importantly, when choosing between different options, surveyors may be able to implicitly manage tradeoffs between survey coverage and the detectability of targets. At one extreme, basic count methods, including quadrat surveys, point counts, and transect surveys are the simplest approaches to implement; however, these methods may require significant effort to ensure every individual is counted, limiting the area that surveyors cover. Furthermore, there are many situations in which it may be impossible to observe all individuals due to logistical constraints and the cryptic nature of many species (Kellner & Swihart, 2014). For example, our past work applying distance survey methods to zebra mussels in Minnesota lakes found that a diver could detect about one out of every four zebra mussels (Ferguson et al., 2019). A variety of methods have been developed to jointly estimate detection and animal density, including mark-recapture (Grimm, Gruber & Henle, 2014), removal (Seber, 1973), distance surveys (Buckland et al., 2001), N-mixture models (Royle, 2004), and sightability surveys (Fieberg, 2012). These approaches may allow a surveyor to move faster and cover more area than quadrat surveys but at the cost of also needing to account for imperfect detection.

In this study, we examined the efficiency of three survey designs in three lakes that had a range of zebra mussel densities. In each lake, we estimated the density of zebra mussels using quadrat, removal, and distance survey methods. Quadrat surveys are assumed to have perfect detection but may be inefficient at lower densities, especially when individuals are clustered (Brown & Manly, 1998); we expect spatial clustering of zebra mussels due to their association with patchy hard substrate and aggregations of attached individuals known as druses (Karatayev, Burlakova & Padilla, 2015). In distance surveys, observers must measure the perpendicular distance of each detection from the transect line. These distances are then used to model how detection probability declines with distance from the surveyor and to estimate density from the observed counts (Buckland et al., 2001). When densities are low, distance surveys may allow users to cover a larger area in a fixed amount of time relative to quadrat surveys. On the other hand, they may be inefficient at higher densities due to the time required to measure the distances between each object and the transect line. Finally, removal surveys (Cook & Jacobson, 1979) utilize a second observer who notes observations missed by a first observer. Removal surveys should require less time than distance surveys to collect the information needed to estimate detection since no distance measurements are required. However, removal surveys will typically have smaller transect widths than distance surveys to ensure detection probabilities are constant and do not vary with distance from the surveyor.

In each lake, we implemented all three survey designs, then compared estimates of zebra mussel density and their associated uncertainties to evaluate the relative performance of the different methods. We predicted that the relative survey efficiency (*i.e*., required sampling effort to achieve a pre-specified level of precision) would depend on the underlying animal density. This dependence would arise due to tradeoffs between survey effort allocated to counting mussels in spatially distinct areas *vs*. the effort required to collect information necessary to estimate detection probabilities. We further predicted that at lower densities, distance and removal surveys would cover more area in a fixed amount of time and would outperform quadrat surveys. By contrast, we expected quadrat surveys to perform best at higher densities since this design does not require additional measurements with each detection event.

## Methods

### Field surveys

**Figure 1:**
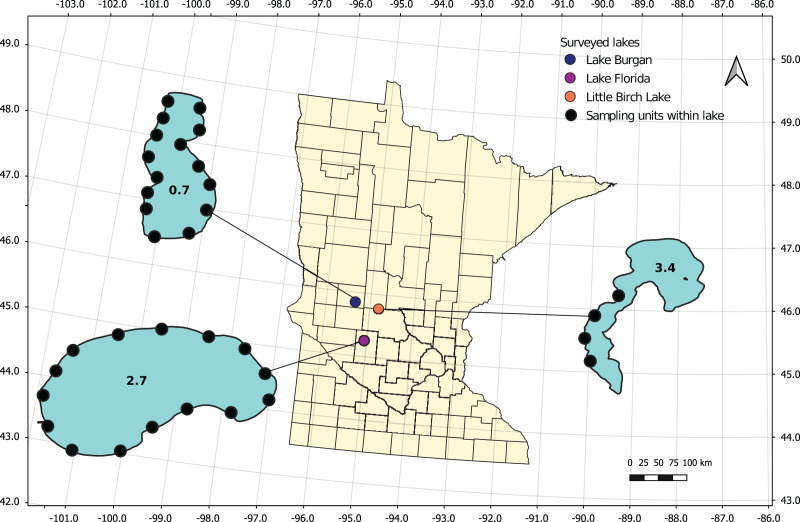
Location of three lakes surveyed in Minnesota during the summer of 2018. Solid black circles within each lake indicate surveyed locations. Lake surface area (in km^2^) is reported within each lake polygon.

In 2018, we conducted exploratory dives in six candidate lakes in central Minnesota (Christmas Lake, East Lake Sylvia, Lake Burgan, Lake Florida, Little Birch Lake, and Sylvia Lake) that have had confirmed recent zebra mussel infestations (as determined by the Minnesota Department of Natural Resources). We established 15 survey sites in each lake, approximately evenly distributed around the lake perimeter using ArcMap, then located the sites in the field using a GPS unit (Garmin GPSMAP 64s). Sites occurred at depths between 0.5 to 4.5 m. At each site, two divers fanned out and spent 20 min underwater counting all mussels they encountered (counts provided in Table S1). We used these initial timed counts to select three lakes with a range of apparent densities to further survey during the summer of 2018: Lake Florida in Kandiyohi County, Lake Burgan in Douglas County, and Little Birch Lake in Todd County (Fig. 1). Lake Florida covers an area of 273 hectares and has a maximum depth of 12 m, Lake Burgan covers an area of 74 hectares and has a maximum depth of 13 m, and Little Birch Lake covers 339 hectares and has a maximum depth of 27 m.

**Figure 2:**
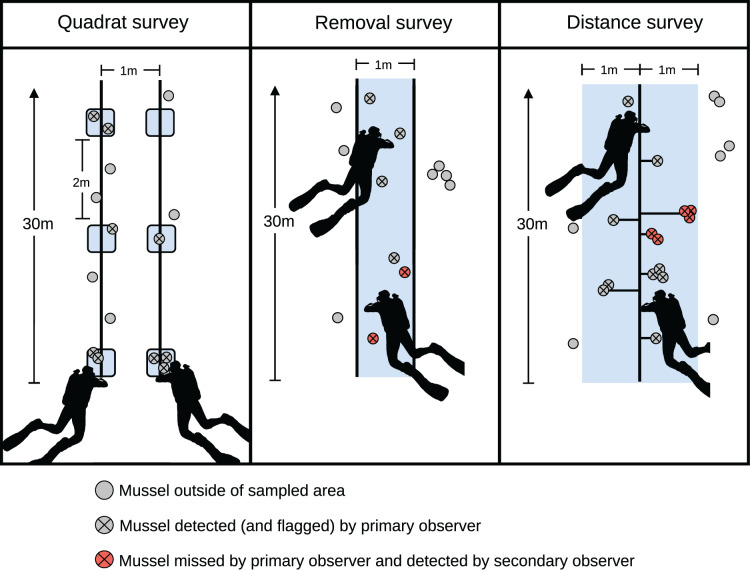
Illustration of a transect for each of the survey techniques used in this study. The blue-shaded area indicates the area surveyed by the dive team. Horizontal lines in the distance survey indicate the distance measures used to estimate detection probabilities.

In each of these three lakes, we surveyed mussels using three methods: quadrat, removal, and distance-removal surveys. For each survey method, we used the site locations from the initial timed survey to determine the start of each survey transect. For quadrat and removal surveys, we laid two parallel 30-m transect lines spaced 1 m apart at each site location, while a single 30-m transect line was used to survey each site with distance-removal surveys (Fig. 2). All transects were placed perpendicular to the shoreline.

We visited Lake Burgan during the second week of July 2018, followed by Little Birch Lake the next week, and Lake Florida the week after. We spent 3 days surveying each lake, with a single day allocated to each survey method, starting with the removal survey on day 1 and ending with the quadrat survey on day 3. A single day was not sufficient to complete all 15 transects in each lake, so we revisited the lakes in August to complete the remaining transects. We observed high densities of newly-settled zebra mussels in Little Birch Lake from a recruitment event between our first and second visits. No other lake had juvenile zebra mussels present at the time of our surveys. Thus, we limited our analyses to adult observations and only considered the four transects completed in July when estimating density in Little Birch Lake (Fig. 1).

For quadrat surveys, each diver on our two-person team surveyed one of the parallel transects, placing a $0.5 \times 0.5$ square-meter quadrat every 2 m along the transect starting at 0 m (Fig. 2). Divers then counted all zebra mussels within the quadrat. For a full 30-m transect, this design resulted in 30 quadrat counts (quadrats were not placed at the 30-m mark since this would result in counting mussels outside of the transect). Both divers sampled an additional 113 quadrats placed along the 11 transects in Little Birch Lake that were not sampled using the other methods due to the recruitment event. Each diver independently sampled these 113 quadrats (hereafter, repeated quadrat counts). The repeated quadrat counts were compared to evaluate the assumption of perfect detection in the quadrat surveys.

To conduct removal surveys, the parallel transect lines allowed divers to determine whether mussels were in the strip-transect (Fig. 2). We used the same GPS locations as the quadrat survey to set up the removal transects, although the exact locations of the transects likely differed slightly since we did not physically mark the start of each transect. The first diver swam between the transect lines and whenever the diver detected a zebra mussel or a cluster of mussels, they marked the location with a survey flag and recorded the number of mussels in the cluster, thereby removing the detected individual or cluster from subsequent counts by the second diver. Each cluster was considered a single independent detection event and the number of individual mussels (hereafter cluster size) in the cluster was recorded. The second diver followed behind after a delay to reduce any turbidity that may have been caused by the first diver. They collected the flags and looked for additional mussels missed by the first diver. Divers alternated between the primary and secondary observer roles between transects to average out any innate differences between observers following the recommendation of Cook & Jacobson (1979).

Lastly, we conducted distance-removal (hereafter, distance) surveys. For these, divers surveyed up to 1 m on either side of the transect (Fig. 2). The first diver marked detections, as in the removal survey, then measured the perpendicular distance from the detection to the transect line. The secondary diver then looked for zebra mussels that were missed by the primary diver. We used this approach rather than conventional distance sampling since we previously found that detection along the transect line was imperfect (Ferguson et al., 2019). As in the removal survey, we treated each detected cluster as an independent detection event. For any of the survey techniques, transects were stopped earlier than 30 m if divers ran into the thermocline due to lowered visibility making detections less reliable.

In addition to the information required to estimate density, we also measured habitat substrate along the transect, water clarity using a Secchi disk, proportional plant cover over the length of the transect, and the depth at the start of our transect following methods described in Ferguson et al. (2019), although this information was not used in subsequent analyses. We also recorded the total time required to complete each transect for each survey design and used these measurements to explore differences in the time required to complete surveys by fitting a linear model to the log (transect survey times) with lake, survey method, and their interaction included as predictor variables. The interaction effect allowed us to determine if the relative cost of completing the surveys (measured in units of time) varied across lakes and survey methods.

#### Density estimates

Let ${n_i}$ denote the number of detections for the ${i^{{\rm{th}}}}$ transect and $n = \sum\nolimits_{i = 1}^T {{n_i}}$ be the total number of detections over all *T* surveyed transects. Further, let $w$ denote the transect width, ${l_i}$ the length of the ${i^{{\rm{th}}}}$ transect, and $L = \sum\nolimits_{i = 1}^T {{l_i}}$ denote the total length of all surveyed transects. We estimated zebra mussel density (in units of individuals/m^2^) using (Buckland et al., 2001):

$\hat D = {{n \cdot \widehat {{\rm{E}}(s)}} \over {2w \cdot \hat P \cdot L}},$

where $\widehat {{\rm{E}}(s)}$ was the estimated average cluster size and $\hat P$ was the estimated average probability of detection. In the case of quadrat surveys, the detection probability was assumed to be 1, and the variance in the detection probability was assumed to be zero. Detection probabilities for distance and removal surveys were obtained using the R package mrds (Laake et al., 2018). For the removal surveys, we used the removal configuration with full independence, which assumes that observations for the two observers are independent; for the distance surveys, we used the removal configuration coupled with the half-normal detection function for the distance model. We evaluated the goodness-of-fit of the detection model using a chi-squared test following Borchers et al. (2006).

The uncertainty in the estimated density can be approximated by Buckland et al. (2001):

${\rm{ Var}}(\hat D) \approx {\hat D^2}\left( {{{{\rm{Var}}(n)} \over {{n^2}}} + {{{\rm{Var}}(\widehat {{\rm{E}}(s)})} \over {{{\widehat {{\rm{E}}(s)}}^2}}} + {{{\rm{Var}}(\hat P)} \over {{{\hat P}^2}}}} \right).$

We used a design-based estimator to determine the variance in the counts (Buckland et al., 2001),

${\rm{ Var}}(n) = {L \over {T - 1}}\sum\limits_{i = 1}^T {{l_i}} {\left( {{{{n_i}} \over {{l_i}}} - {n \over L}} \right)^2},$

where the contribution of each transect to the total variance was weighted by the transect length, ${l_i}$. The uncertainty in the average cluster size, $V(\widehat {{\rm{E}}(s)})$ was calculated as the sample standard error in the average cluster size following (Buckland et al., 2001). For the removal and distance surveys, the uncertainty in detection, ${\rm{Var}}(\hat P)$, was reported by the mrds package.

We estimated the number of transects needed to achieve a coefficient of variation (hereafter CV) of 0.1 (Buckland, 2006), denoted as ${T_{{\rm{estimated}}}}$, by solving the equation $0.1 = {\rm{CV}}(\hat D){{{T_{{\rm{completed}}}}} \over {{T_{{\rm{estimated}}}}}}$, where CV( $\hat D$) is the CV we obtained from our original survey with ${T_{{\rm{completed}}}}$ transects. For Lake Florida and Lake Burgan, ${T_{{\rm{completed}}}}$ was 15 transects, whereas ${T_{{\rm{completed}}}}$ was four for Little Birch Lake (Table 1).

**Table 1:** Summary of quadrat, removal, and distance surveys performed in three Central Minnesota lakes surveyed during the summer of 2018. We report the total area surveyed, the number of detected clusters, and the total time taken to complete all the transects in each survey. CV denotes the coefficient of variation in the estimated density.

Lake surveyed	Method	Transects	Area surveyed ( ${m^2}$)	Detections	Total survey time (h)	CV
	Distance	15	900.0	10	3.8	0.38
Lake Florida	Removal	15	450.0	5	4.46	0.41
	Quadrat	15	112.5	8	4.4	0.42
	Distance	15	602.0	79	6.1	0.05
Lake Burgan	Removal	15	277.0	47	5.8	0.10
	Quadrat	15	71.5	40	5.3	0.14
	Distance	4	124.0	252	5.9	0.25
Little Birch Lake	I Removal	4	84.0	152	4.1	0.22
	Quadrat	4	21.5	526	2.6	0.14

## Results

**Figure 3:**
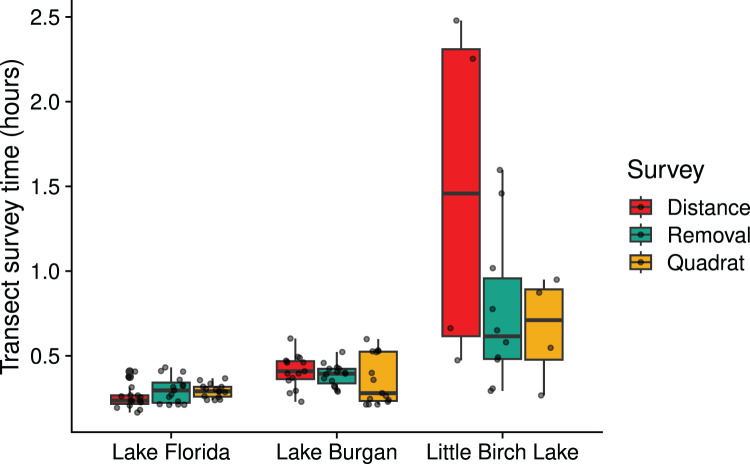
Boxplot indicating the amount of time spent surveying a transect for distance, removal, and quadrat surveys in three Central Minnesota lakes surveyed during the summer of 2018. The lower and upper hinges denote the first and third quartiles, and the horizontal line denotes the median. Points indicate the individual data points.

Across the three lakes, we covered the most area using distance surveys (1,626 m^2^), compared to 811 m^2^ using removal surveys, and 205.5 m^2^ using quadrat surveys (Table 1). When modeling transect survey times (Fig. 3), we found evidence that lake and survey design interacted to influence the median time to survey transects (F = 3.1845, df $_1 = 99$, df $_2 = 4$, *p* = $0.002$). Residual plots provided reassurance that model assumptions (normally distributed errors with constant variance) were reasonably met. In Lake Florida, our lowest-density lake, the median transect survey time was lowest for distance surveys, while removal and quadrat surveys had similar median survey times. In Lake Burgan and Little Birch, Lake the median transect survey time was lowest for quadrat surveys and highest for distance surveys, with removal surveys falling closer to distance surveys than quadrat surveys. Estimated median transect survey times are provided in Table S2.

In Little Birch Lake, repeated quadrat counts by the two observers were highly correlated ( $\hat \rho = 0.83$) but were not identical even when the number of observed mussels was small (*e.g*., $\lt5$; Fig. S1). However, these counts occurred after the reproductive event in Lake Florida and may have been influenced by the newly recruited mussels, which are small and more difficult to detect than adults. We discuss the potential implications of ignoring measurement error in the quadrat counts in the discussion.

### Density estimates

**Figure 4:**
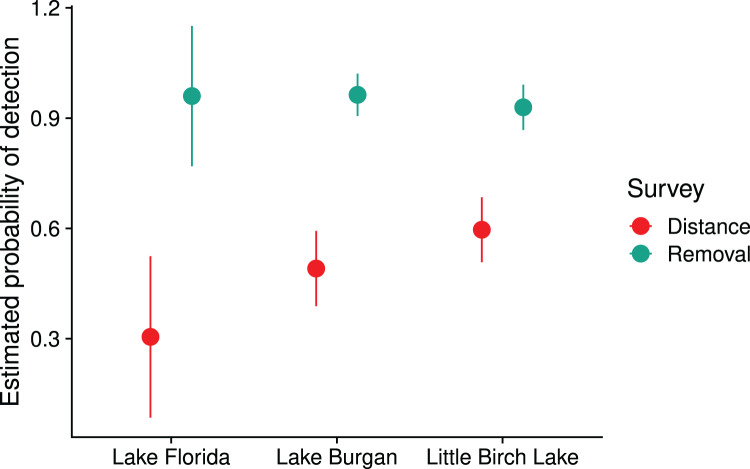
Estimated probability of detection, $\hat P$, for removal and distance surveys in three Central Minnesota lakes surveyed during the summer of 2018; detection probabilities were assumed to be one for quadrat surveys. Error bars denote two standard errors.

We did not detect a lack of fit of the half-normal detection model when analyzing the data from Lake Burgan ( ${\chi}{^2} = 2.82$, $k = 3$, *p* = 0.42) or Little Birch Lake ( $\chi{^2} = 40.54$, $k = 7$, *p* = 0.16); we did not have sufficient data to run the test using the data from Lake Florida. Our estimated probabilities of detection were lower in the distance surveys than in the removal surveys (Fig. 4). The probability of detection in the removal surveys was consistently estimated above 0.9, whereas estimates ranged from about 0.3 to 0.6 in the distance surveys. In Lake Burgan and Little Birch Lake, the standard errors of the detection probabilities were slightly lower in the removal surveys than the distance surveys, despite removal surveys producing about half the detection events of a distance survey conducted in the same lake (Table 1).

**Figure 5:**
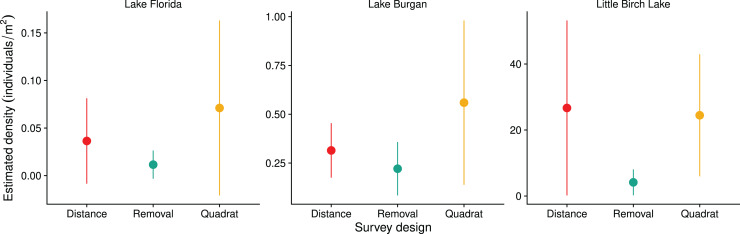
Density estimates (individuals per m^2^) for quadrat, removal, and distance surveys in three Central Minnesota lakes surveyed during the summer of 2018. Error bars denote two standard errors.

Consistent with our initial timed searches on these lakes, we found that Lake Florida had the lowest estimated density, Lake Burgan had an intermediate estimated density, and Little Birch Lake had the highest estimated density (Fig. 5). Removal surveys always resulted in the lowest estimated densities. Our application of this method assumed that detection probabilities were constant for all mussel clusters. When the true detection probabilities are heterogeneous (*e.g*., dependent on cluster size or the distance between the cluster and the observer), estimated detection probabilities will be biased high and estimates of density biased low (Caughley & Grice, 1982). This may explain the slightly lower estimates of density using the removal method. Estimates from distance surveys correct for heterogeneity in detection due to distance but could be impacted by heterogeneity associated with cluster size.

Lake Florida had the highest CV for all survey designs (Table 1). In both Lake Florida and Lake Burgan, quadrat surveys had the highest CV followed by removal and then distance surveys. However, in Little Birch lake this trend was reversed with distance surveys having the highest CV followed by removal and then quadrat surveys.

All mussel detections in Lake Florida were of single zebra mussels, whereas in Lake Burgan, the average cluster size in the removal survey was $\widehat {{\rm{E}}(S)} = 1.25$ (SE = $0.12$) and 1.17 (SE = $0.05$) in the distance survey. Clusters were largest in Little Birch Lake, with an average cluster size in the removal survey of $\widehat {E(S)} = 4.81$ (SE = $0.47$) and $7.83$ (SE = $0.92$) in the distance survey. In the distance survey, we found five clusters with more than 66 individuals, twice the maximum cluster size found in the removal survey. Differences in the distribution of cluster sizes between the two methods may be attributable to sampling slightly different areas due to measurement error associated with locating the transect using the GPS unit and wider transect width in the distance surveys.

**Figure 6:**
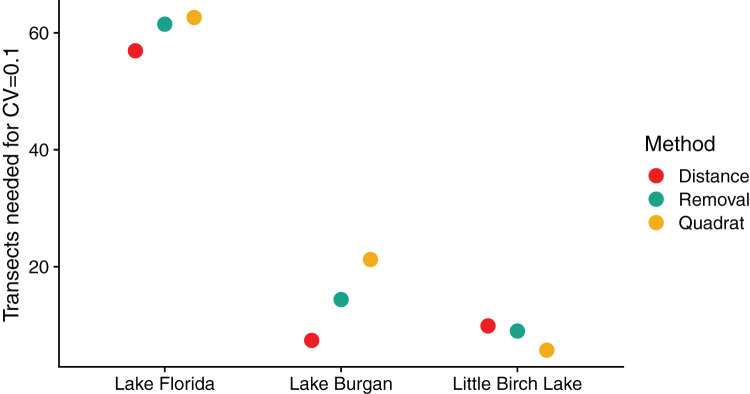
The estimated number of transects needed to achieve a coefficient of variation (CV) of 0.1. Surveys were conducted in three Central Minnesota lakes during the summer of 2018.

The estimated number of transects needed to achieve a ${\rm{CV}} = 0.1$ (Fig. 6) was highest in Lake Florida, the low-density lake, requiring about 60 transects to achieve this goal. The distance survey performed best in this lake (57 transects) followed by the removal survey (61 transects) and quadrat survey (63 transects) (Fig. 6). In Lake Burgan and Little Birch Lake, the number of transects needed was less than half that of Lake Florida, indicating that all survey methods were more efficient in higher-density lakes. In Lake Burgan, the distance survey (Seven transects) performed better than the removal survey (14 transects) and the quadrat survey (21 transects), and in Little Birch Lake the quadrat survey performed best (Six transects) with the distance (10 transects) and removal (Nine transects) surveys performing similarly.

Finally, we examined the proportion of the total variance in the estimated density that was due to uncertainty in detection ( ${\rm{Var}}(\hat P)/{\rm{Var}}(\hat D)$). In all lakes, this proportion was small (ranging from 0.01% to 4%), and the contribution was always lower in the removal surveys than in the distance surveys. Thus, uncertainty in detection had a low contribution to the total uncertainty in density.

## Discussion

We found that the relative efficiency of the three survey methods, in terms of the number of survey transects needed to achieve a ${\rm{CV = 0}}.{\rm{1}}$, varied by lake. In our low- and medium-density lakes, distance surveys required the fewest transects, followed by removal, then the quadrant surveys. In the high-density lake, this pattern was reversed with quadrat surveys being most efficient followed by removal surveys and then distance surveys. These patterns were consistent with our *a priori* predictions that at lower densities we should select survey designs that cover more area at the cost of lower detection rates, while at higher densities these designs become prohibitive due to the cost of making additional measurements with each detection event.

The improved performance of the quadrat survey at the highest densities in our study, relative to the other designs, was likely due to the amount of time it took to record the extra information necessary for estimating detection probabilities. It may be possible to improve the efficiencies of distance and removal surveys by collecting this additional information for a subset, rather than all transects (*e.g*., Pollock et al., 2002). One caveat of this approach would be that the detection of many animals is known to be habitat-specific (*e.g*., Bacheler et al., 2014; Hagihara et al., 2018; Ferguson et al., 2019); thus, the transects used to estimate detection should be representative of the available habitat. Work by Knights et al. (2021) has shown that this could be addressed in distance surveys by measuring only a proportion of the targets, where the optimal proportion can be determined when the search time and the time taken to make a detection are known.

One potential advantage of distance surveys is that the transects can be wider than in removal surveys, which could result in more detections and improved estimates of detectability. However, uncertainty in the detection probabilities contributed little to the variance of $\hat D$, and thus the standard errors were similar for the two methods despite having far fewer detections in the removal survey. Although the distance and removal surveys performed similarly in our study lakes, distance surveys may outperform removal surveys when densities are low and the transect width can be much wider than the width of a comparable removal survey.

Finally, quadrat surveys typically assume perfect detection in each quadrat. Here, we found discrepancies between two observers’ counts when surveying the same quadrat, suggesting the presence of measurement error. While we did not account for this error in our analyses, methods do exist for using repeated counts to jointly estimate population size and the probability of detection that could be explored in future surveys (Royle, 2004). The impact of measurement error on the density estimates will depend on the details of the observation process. Our quadrat density estimates were consistent with the other survey methods (Fig. 5), suggesting that observers were roughly equally likely to over- or under-count the number of individuals. In this case, we expect the variance in the estimated density to account for both the variance in the counts plus additional variation due to the measurement error, leading to higher overall uncertainty in our density estimate than we would obtain without measurement error.

Our estimated detection probability from the distance survey in Lake Burgan, $\hat P = 0.54$ (SE = $0.05$) was comparable to the detection probability we estimated for one of our dive teams from the previous field season, $\hat P = 0.41$ (SE = $0.08$) (Ferguson et al., 2019). Yet, it was substantially higher than our estimate of detection for the other dive team, $\hat P = 0.10$ (SE = $0.07$) (Ferguson et al., 2019), highlighting the importance of accounting for observer-specific detection probabilities. There are a number of examples of differences in detection probabilities between observers in surveys of marine mammals (Laake et al., 1997; Bröker et al., 2019), birds (Nichols et al., 2000; Koneff et al., 2008), and plants (Moore et al., 2011; Alexander et al., 2012). This body of work reinforces the general need to account for observer-specific probabilities when surveys are performed by multiple individuals. Despite the large differences in estimated detection probabilities between years in Lake Burgan, the estimated densities were remarkably similar, with $\hat D = 0.24$ (SE = $0.08$) in 2017 and $\hat D = 0.27$ (SE = $0.06$) in 2018, indicating that one can obtain consistent estimates when controlling for observer-specific detection probabilities.

Although comparisons among survey methods using empirical data, such as was done in this study, can illustrate general patterns in efficiency tradeoffs, the details of these tradeoffs are likely to be highly context-dependent. More general conclusions can sometimes be generated using an analytical framework that captures the costs and efficiencies of different survey methods (*e.g*., Giudice et al., 2010). We are currently developing a framework that links survey efficiency to measures of effort necessary to perform tasks within each survey (*e.g*., the amount of time to set up and survey a transect and the additional time required for each detection event). We hope this framework will provide further insights into the tradeoffs between surveyor effort and detection probabilities, and may also prove useful for optimizing this aspect of survey design.

## Supplemental Information

10.7717/peerj.15528/supp-1 Supplemental Information 1 Timed count searches. Average counts in 30 timed searches (15 min each) for six lakes surveyed in Minnesota. Two observers conducted the surveys and visited 15 transects along the perimeter of the lake. Survey results were used to identify three early invaded lakes for subsequent surveys that covered a range of population densities.

10.7717/peerj.15528/supp-2 Supplemental Information 2 Survey times. Estimated median transect survey time (in hours) and the associated standard errors.

10.7717/peerj.15528/supp-3 Supplemental Information 3 Repeated quadrat counts. Counts from Diver 1 and Diver 2 on repeated quadrats in 11 transects from Little Birch Lake. The dotted line represents the ideal case where no observational error is present in the counts.

## Additional Information and Declarations

### Data Availability

The following information was supplied regarding data availability: The data and code are available at the Digital Repository of the University of Minnesota: Ferguson, Jake M; Jimenez, Laura; Keyes, Aislyn A; Hilding, Austen; McCartney, Michael A; St. Clair, Katie; Johnson, Douglas H; Fieberg, John R. (2023). R Code and Data Supporting: A comparison of survey method efficiency for estimating densities of Zebra Mussels (*Dreissena polymorpha*). Retrieved from the Data Repository for the University of Minnesota, https://doi.org/10.13020/bjdp-p977.
