# Supplementary material for: A comparison of survey method efficiencies for estimating densities of zebra mussels (Dreissena polymorpha)
Source: PeerJ. 2023 Jul 10;11:e15528. doi: 10.7717/peerj.15528 (PMC10340101; doi:10.7717/peerj.15528)
Supplement: Supplemental Information 1 — Average counts in 30 timed searches (15 min each) for six lakes surveyed in Minnesota. Two observers conducted the surveys and visited 15 transects along the perimeter of the lake. Survey results were used to identify three early invaded lakes for subsequent surveys that covered a range of population densities. [file peerj-11-15528-s001.pdf]

Table S1: Average counts in 30 timed searches (15 minutes each) for six lakes surveyed in Minnesota. Two observers conducted the surveys and visited 15 transects along the perimeter of the lake. Survey results were used to identify three early invaded lakes for subsequent surveys that covered a range of population densities.

Lake surveyed	Average counts
Lake Florida	0.3
Lake Burgan	1.7
Little Birch Lake	55.1
East Lake Sylvia	97.7
Lake Sylvia	119.5
Christmas Lake	Uncountable
