# Supplementary material for: A comparison of survey method efficiencies for estimating densities of zebra mussels (Dreissena polymorpha)
Source: PeerJ. 2023 Jul 10;11:e15528. doi: 10.7717/peerj.15528 (PMC10340101; doi:10.7717/peerj.15528)
Supplement: Supplemental Information 2 — Estimated median transect survey time (in hours) and the associated standard errors. [file peerj-11-15528-s002.pdf]

Table S2: Estimated median transect survey time (in hours) and the associated standard errors.

Lake	Design	Median	Standard error
	Distance	0.40	0.04
Lake Burgan	Removal	0.36	0.03
	Quadrat	0.33	0.03
	Distance	1.15	0.20
Little Birch Lake	Removal	0.66	0.15
	Quadrat	0.59	0.10
	Distance	0.25	0.02
Lake Florida	Removal	0.25	0.03
	Quadrat	0.29	0.03
