# Supplementary figures and images for: A comparison of survey method efficiencies for estimating densities of zebra mussels (Dreissena polymorpha)
Source: PeerJ. 2023 Jul 10;11:e15528. doi: 10.7717/peerj.15528 (PMC10340101; doi:10.7717/peerj.15528)

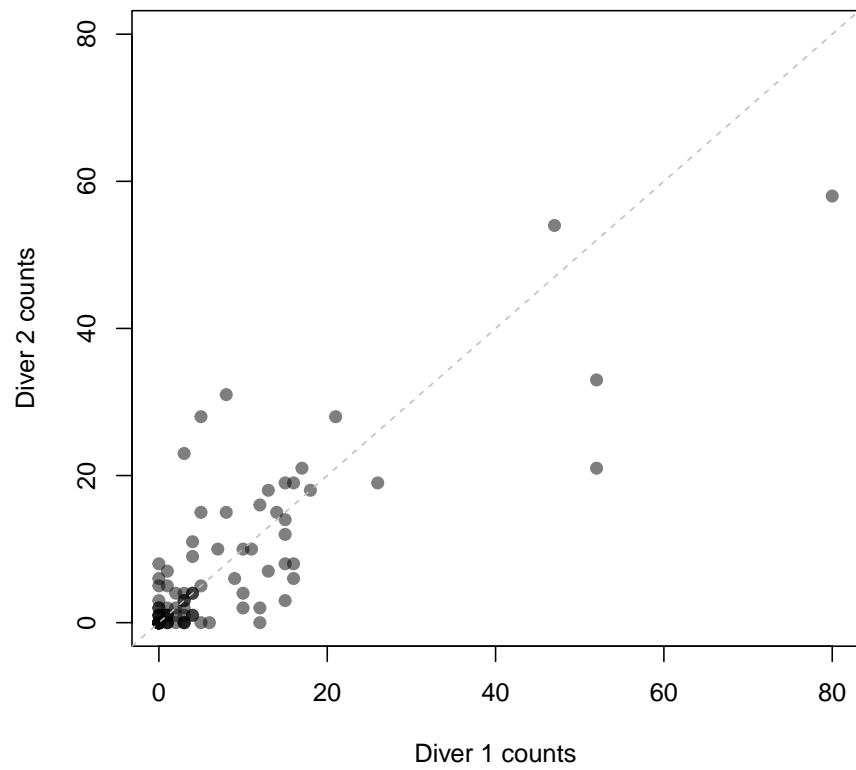

Supplement: Supplemental Information 3 — Counts from Diver 1 and Diver 2 on repeated quadrats in 11 transects from Little Birch Lake. The dotted line represents the ideal case where no observational error is present in the counts. [file peerj-11-15528-s003.pdf]
